# Retrograde coiled spring jejunal intussusception in an infant due to Foley catheter gastrostomy: a case report

**DOI:** 10.1186/s12876-022-02336-4

**Published:** 2022-06-03

**Authors:** Feride Mehmetoğlu

**Affiliations:** Department of Pediatric Surgery, Dortcelik Children’s Hospital, 16140 Bursa, Turkey

**Keywords:** Coiled spring jejunal intussusceptions, Foley catheter migration, Stamm gastrostomy, Prematurity, Case report

## Abstract

**Background:**

For infants who need long-term enteral feeding but are unable to maintain sufficient oral intake, feeding gastrostomy tube placement is required. The use of a Foley catheter as a replacement catheter in a Stamm gastrostomy is indicated in the absence of dedicated gastrostomy feeding tubes; however, this approach has been associated with many morbidities. In this report, an unusual case of an infant who underwent a major operation due to coiled spring jejunal intussusception caused by Foley catheter migration is described.

**Case presentation:**

A 6-month-old neurologically impaired premature female patient was admitted to the emergency unit with respiratory distress, nonbilious vomiting and an ineffective gastrostomy feeding tube. Her history revealed that, at the age of 2 months, she had undergone Stamm gastrostomy for enteral feeding with a Pezzer catheter. However, at the age of 5 months, the Pezzer catheter became dislodged and was replaced with a Foley catheter. The patient subsequently underwent emergent exploratory laparotomy due to intestinal obstruction. During the operation, retrograde coiled spring jejunal intussusceptions with multiple areas of local necrosis and perforations were observed. Resection of the affected jejunal segment and end-to-end anastomosis were performed. The postoperative period was long and very demanding due to the presence of several comorbidities. To our knowledge, this is the first operative demonstration of coiled spring intussusception.

**Conclusion:**

This case report aims to increase clinical awareness of the possibility of coiled spring intussusception following the use of Foley catheter in a gastrostomy and the difficulties encountered in the surgical course of a premature infant.

## Background

Infants who have congenital anomalies, respiratory insufficiency, neurologic injury or gastrointestinal conditions that affect their oral feeding ability require gastrostomy tube placement for long-term enteral feeding [1]. Complications related to feeding tube placement in children are common and can range from minor to life-threatening [2] and may be related to the surgical procedure or the catheter being inserted [3]. Pezzer or Foley catheters are used as gastrostomy tubes with the Stamm technique in cases where special gastrostomy catheters are not available and percutaneous or laparoscopic gastrostomy is not an option [4–6].

Although Foley catheters are indicated for urinary catheterization, their use as a replacement catheter for gastrostomy feeding tubes is relatively common [7, 8]. However, the effectiveness, safety and ethical considerations of using a Foley catheter as an enteral feeding tube remain highly debated [4, 7]. The use of Foley catheters as gastrostomy tubes can result in certain complications; for example, the balloon may rupture, allowing the catheter to slip out, and distal migration of the tube can occur, which could potentially lead to obstruction of the gastric pylorus or small bowel [9, 10]. Antegrade-peristaltic intussusception and retrograde-antiperistaltic intussusception have been very rarely reported as complications of gastrostomy tube migration, and the exact mechanism leading to intussusception is unknown [3]. Thus, education of the caregiver/patient is essential to prevent these complications due to catheter mobilization [11].

In this report, a rare case of a premature infant who developed retrograde coiled spring jejunal intussusception after Foley gastrostomy is presented.

## Case presentation

A female infant weighing 1790 g was born to a 35-year-old gravida 2 para 2 mother with an unremarkable family history by emergency cesarean section at a tertiary referral hospital because of decreased fetal movement and oligohydramnios at 32 weeks of gestation. After birth, the infant was transferred to the neonatal intensive care unit for the management of respiratory distress syndrome and prematurity. At 10 days of age, she was discharged home with follow-up instructions.

**Fig. 1:**
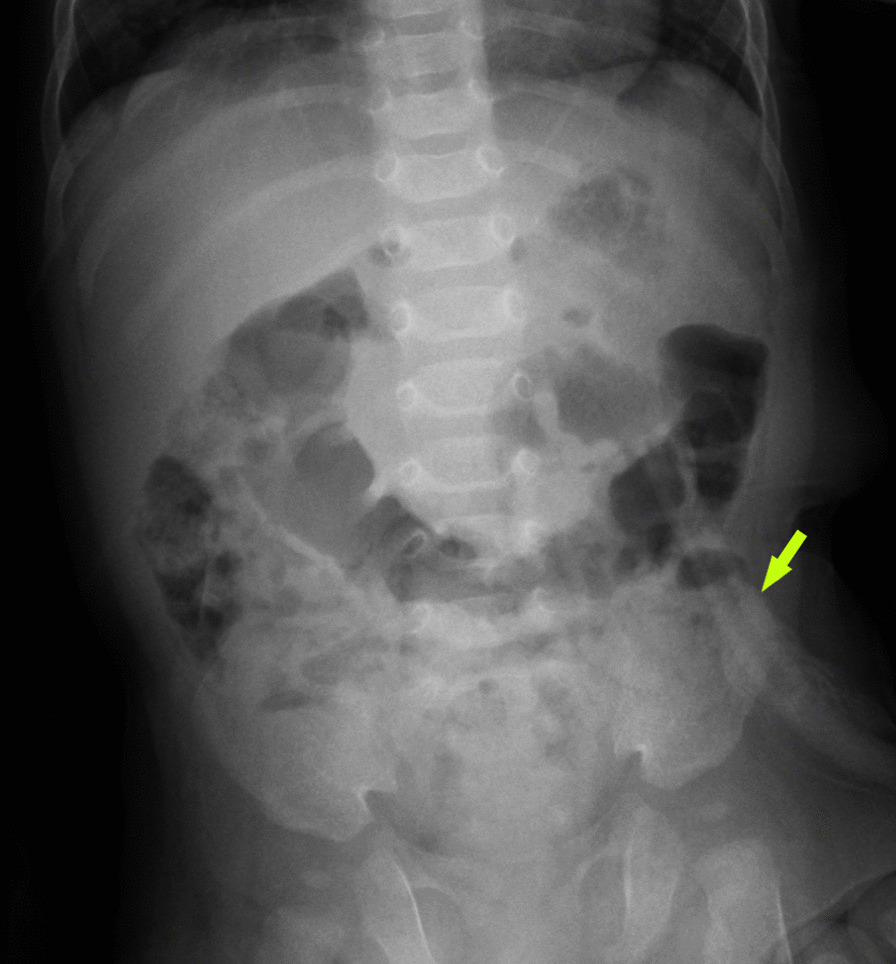
Upright abdominal X-ray of the Pezzer catheter (green arrow) 4 months after placement

At 18 days of age, a serious aspiration event occurred following breastfeeding. The infant was resuscitated by the ambulance crew at home and transferred to our hospital for respiratory ventilator support. Unfortunately, after the third day, sepsis and seizure activity developed. Following 30 days of treatment, she recovered clinically. However, due to neurologic impairment secondary to pulmonary aspiration asphyxia, she was unable to maintain sufficient oral intake. The decision to place a gastrostomy feeding tube was made, and the patient underwent a Pezzer catheter gastrostomy with the Stamm technique at 2 months of age. She was then discharged home, with a weight of 2800 g. At 4.5 months of age, the patient was admitted to our hospital with mild vomiting complaints, weighing 5100 g. The patient was hospitalized for 1 day due to prematurity and comorbidities. The physical examination and diagnostic workup were normal. Upright abdominal X-ray (UAXR) showed that the transverse colon frame was gaseous and that the Pezzer catheter remained in place (Fig. 1).

**Fig. 2:**
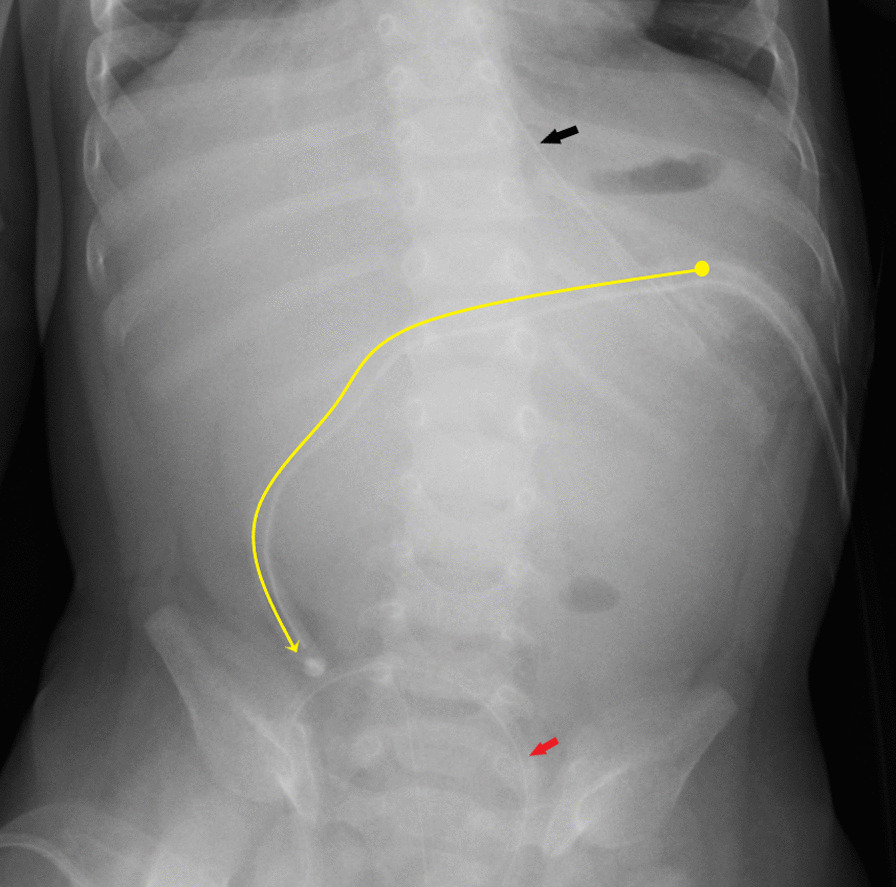
Upright abdominal X-ray the Foley catheter 1 month after Pezzer catheter replacement. The intraabdominal part of the Foley catheter is indicated (yellow line). Nasogastric tube (black arrow) and bladder catheter (red arrow)

**Fig. 3:**
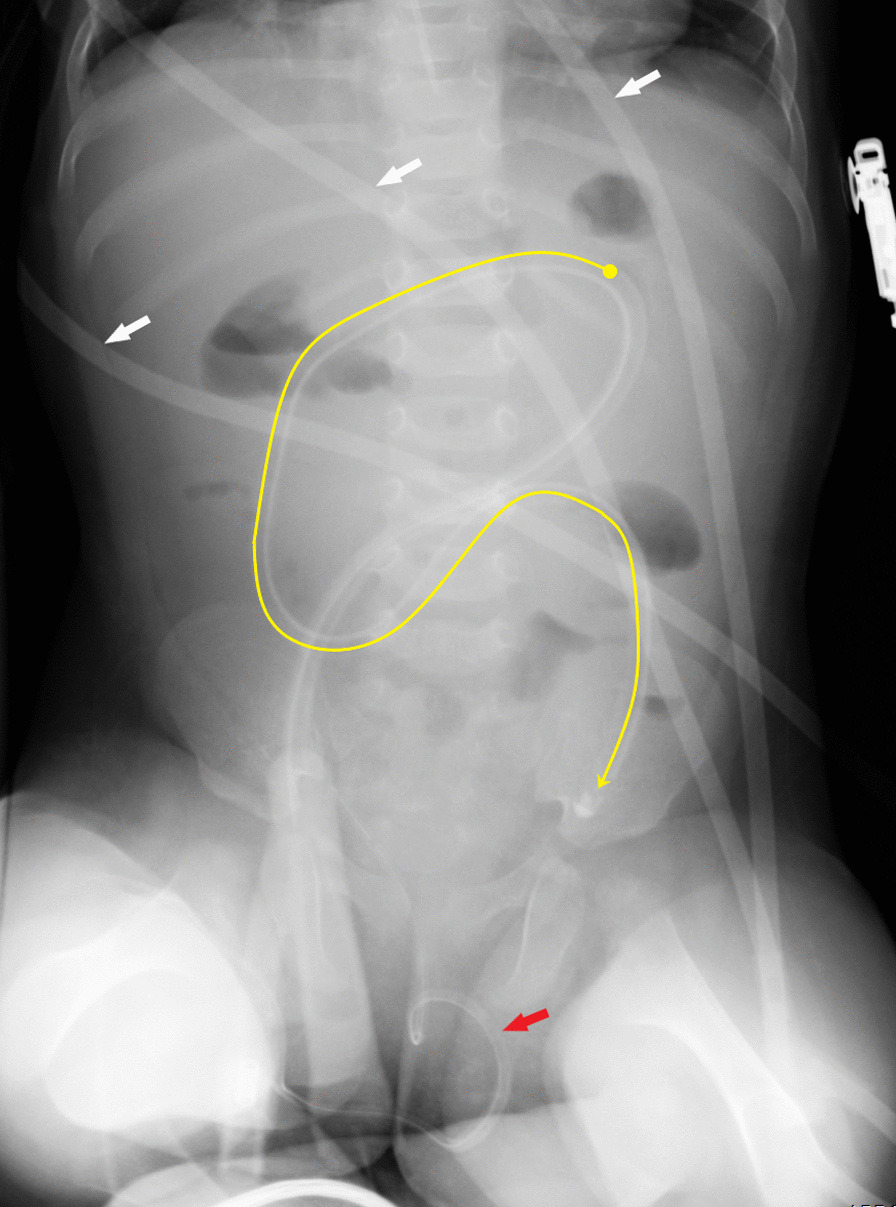
Upright abdominal X-ray of the patient showing the Foley catheter migrating forward in the pelvis and air-fluid levels. The intraabdominal part of the Foley catheter is indicated (yellow line). Intravenous lines (white arrows), bladder catheter (red arrow)

At 5 months of age, the Pezzer tube was dislodged unintentionally at home, and a 12-French silicon Foley catheter was inserted at a local hospital. At 5 months and 3 weeks of age, the patient was admitted again to our hospital with respiratory complaints, nonbilious vomiting, and ineffective Foley catheter gastrostomy. The mother said she tried breastfeeding over the past few days, but the infant could not tolerate it well and would begin to cough. A chest exam and chest X-ray revealed pneumonia. Her abdominal exam was unremarkable: her abdomen was soft, mildly distended, had no palpable mass, and had normal bowel sounds. A rectal examination revealed normal stool. On UAXR, it was found that the abdomen was gasless and that the Foley catheter extended to the pelvis (Fig. 2). On the second day of hospitalization, the patient's condition deteriorated rapidly, requiring transfer to the intensive care unit and ventilation. On the fourth day, the patient developed septic shock, and bilious drainage from her gastrostomy catheter was observed. UAXR revealed that the Foley catheter had moved even further distally and demonstrated air-fluid levels, suggestive of intestinal obstruction (Fig. 3). Although her abdominal examination remained unremarkable, abdominal ultrasonography identified a small bowel obstruction with peritoneal free fluid, and the patient passed a small amount of bloody stool after a 0.9% sodium chloride saline solution enema. Only bedside studies could be performed due to her extensive comorbidities.

**Fig. 4:**
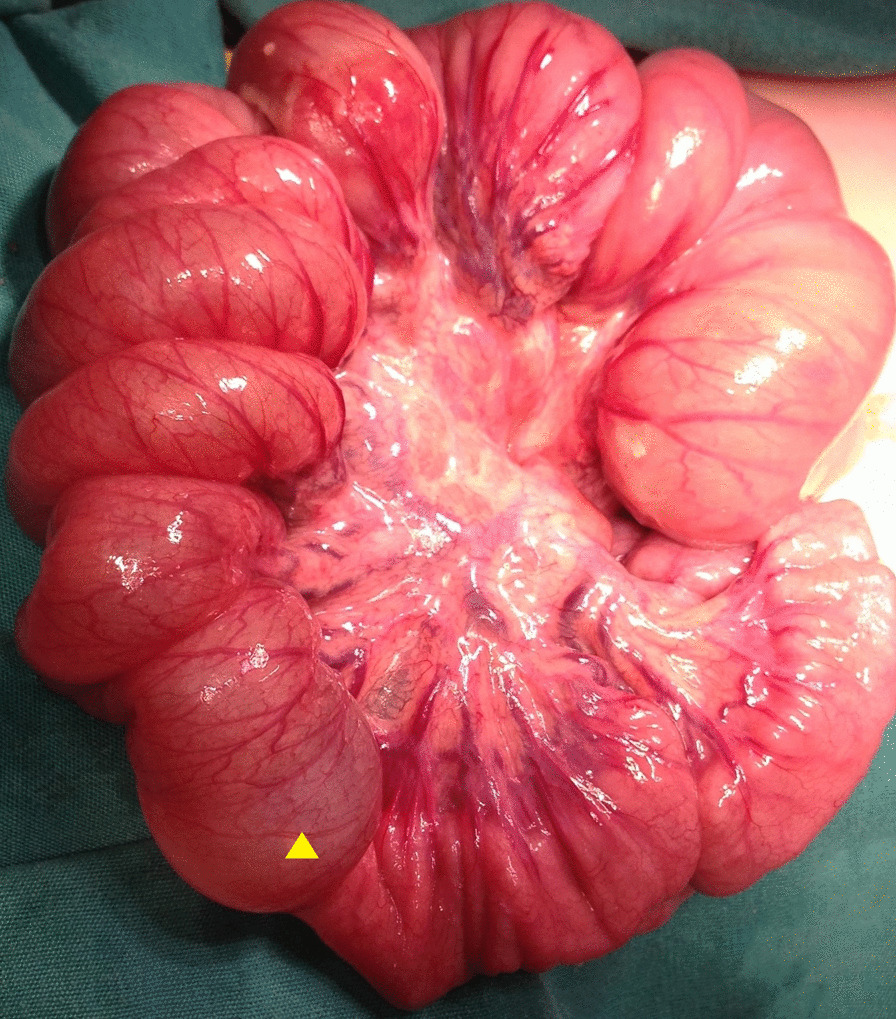
Operative view of intussusceptions with approximately ten loops. Sterile water-filled Foley catheter balloon (yellow arrow)

**Fig. 5:**
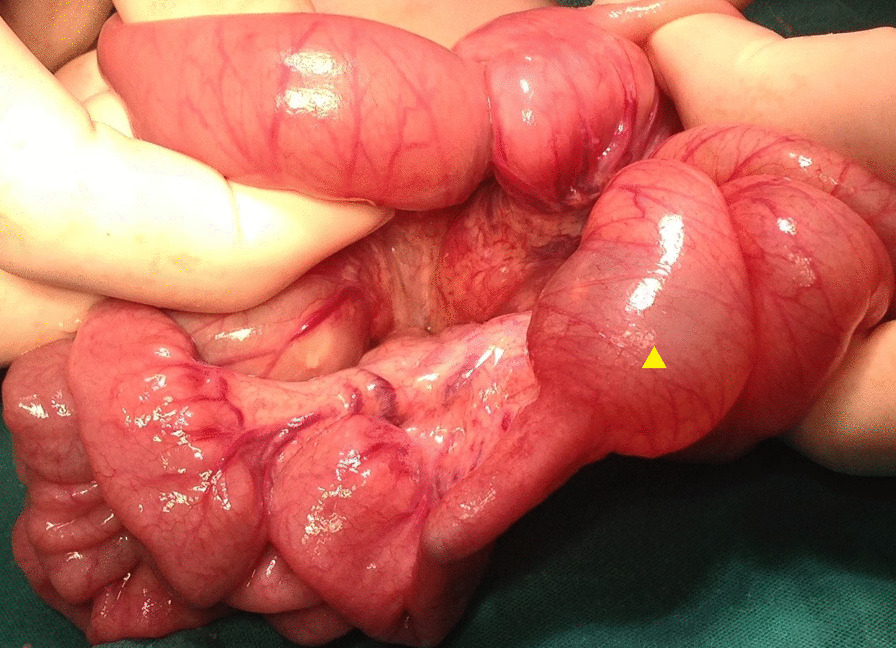
One of the intussusceptions before reduction. Sterile water-filled Foley catheter balloon (yellow arrow)

**Fig. 6:**
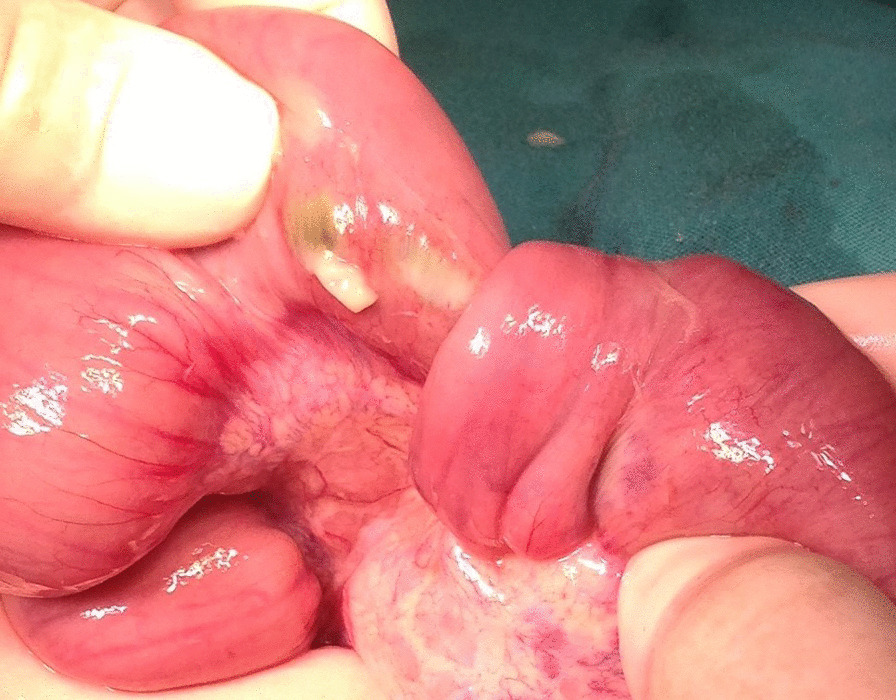
One of the intussusceptions during reduction shows a necrotic area with minute perforation

Laparotomy was indicated according to the patient’s imaging results and clinical findings, with an initial diagnosis of intestinal obstruction. Dilated jejunal bowel loops that formed multiple consecutive jejunal intussusceptions were detected during laparotomy. The Foley catheter had migrated forward from the stomach to the mid-small intestine—a distance nearly 10 times its length. Retrograde intussusceptions involving 70 cm of jejunum were detected, which were formed by approximately 10 separate 5–6 cm intussusceptions starting from the Ligament of Treitz (Fig. 4), and manual reduction was performed (Fig. 5). Careful evaluation of the reduced jejunal segments showed that most of the intussuscepted segments had necrotic areas of up to 1–2 cm, with minute perforation near the mesenteric border (Fig. 6). The affected jejunal segment was resected and anastomosis performed. Postoperatively, the patient returned to the intensive care unit and remained intubated. She was discharged with 12 Fr gastrostomy tube (Avanos Medical Devices), 6 weeks after admission.

## Discussion and conclusions

Foley catheters, which were designed for urinary drainage approximately a century ago, have a wide range of indications in the gastrointestinal system [7]. However, the use of a Foley catheter as a feeding gastrostomy catheter can cause secondary coiled spring retrograde intussusception. The exact mechanisms of feeding tube-induced antegrade and retrograde intussusceptions are unknown and may be distinct for each type. In cases of antegrade intussusception, the feeding catheter irritates the bowel and causes thickening, which then serves as a lead point, or the balloon acts as a lead point. In rarer cases of retrograde intussusception, retraction attempts to retrieve a migrated catheter, similar to a fishing rod, can result in intussusception of the distal bowel into the proximal bowel and cause retrograde intussusception, as in our patient [3]. While the etiology of intussusception in the pediatric population is mostly idiopathic, secondary intussusception can occur due to a variety of etiological factors [12, 13]. The inclusion of feeding catheter migration as a rare cause of secondary intussusception in children will be helpful for early diagnosis.

Feeding catheter-related antegrade and retrograde intussusception studies are mainly presented as case reports. The interval time from tube placement to intussusception differs for each case, ranging from a few days to years and there is no specific at-risk age group. Different lengths of intussusceptions involving the stomach, duodenum, jejunum and ileum due to feeding catheters that were initially inserted or replaced have been reported. Different types/brands of feeding tubes, including PEG (percutaneous endoscopic gastrostomy) and Pezzer catheters, via open or endoscopic approaches have also been reported. Given the limited number of patients in the literature, it is not possible to identify the etiologic or risk factors for intussusception. [3, 13–21].

Three adult patients with different lengths of retrograde intussusceptions involving the stomach, duodenum and jejunum were published as case reports [16–18]. Our patient is the youngest case of retrograde intussusception reported in the literature.

Intussusception due to a feeding tube is diagnosed rather late despite physical examination and imaging test results because classical intussusception findings are not physically observed, and catheter-related intussusceptions are more clinically stable than classic intussusception, except for bilious vomiting [15]. Inconsistent with ileus features, the abdomen can be scaphoid [3] and soft with normal bowel sounds [16]. The feeding tube drainage contents can also be a diagnostic tool [13]. The diagnosis of intussusception can be made with water soluble contrast studies, which may show a coiled spring appearance [15, 16], with air when administered through the feeding tube fluoroscopically [20], or by abdominal ultrasound, (US) [19], contrast computed tomography scan [13], or upper endoscopy [17]; a diagnosis can also be made postmortem [14].

A variety of approaches can be used to reduce intussusception and to evaluate bowel viability. Some patients are first evaluated/diagnosed via endoscopy or laparoscopy and then converted to open surgery [13, 18]. Surgical management techniques include manual reduction [13], repairing only bowel perforations post-reduction [16], or resection and anastomosis of the affected segment [18, 19]. Other treatment options for patients with feeding tube-related intussusception who have a better health status or who have significant surgical risks include close observation after removal or replacement of the gastric tube [17, 21]. However, these options are not always sufficient, especially for small infants with neurologic and comorbid disease, because there is the possibility of ischemic bowel within the intussusception [19]. Additional investigations to rule out intussusception are warranted.

We want to emphasize that multiple minute perforations are specific to multiple consecutive intussusceptions. Ragunath et al. [16] detected three separate minute perforations, whereas we detected seven. Especially in the setting of multiple consecutive intussusceptions, once the bowel is reduced, each intussuscepted segment should be evaluated for minute perforations. Since all infants with feeding tubes have one or more serious diseases that affect oral feeding, a difficult diagnosis and postoperative period should be expected [13, 19].

The shortening of the external portion of the catheter may be an early indication of migration even before clinical evidence of small bowel obstruction can be found [16]. After the operation, the mother of the patient in our case stated that the external part of the catheter had appeared shorter during the past few days and that she tried to pull it back into place. If the mother knew the risks of displacement of the gastrostomy tube, early warning would probably have reduced the severity of the multiple consecutive intussusceptions. Education of caregivers and providing written and verbal instructions well before discharge are important to avoid complications due to catheter migration, even in those who have an external anchoring device.

In a study exploring the ideal gestational age for feeding tube placement for reliable nutrition after discharge, it was found that patients at < 30 w gestational age could undergo feeding tube placement, mostly for complications of prematurity [1]. It was thought that the first aspiration leading to intubation could have been prevented if a feeding catheter had been inserted before discharge in our patient, who was 32 weeks of gestation.

The placement of gastrostomy devices has increased over time, and a Foley catheter is placed in a patient at least once during initial insertion or replacement [4, 22]. Foley catheter complications therefore increase in parallel with the number of tube-fed patients. Although minor and major complications occur with all types of catheters [2], the use of Foley catheters for enteral nutrition can cause many problems in terms of the use of unlicensed products and due to professional and ethical responsibilities [4]. Moreover, there are no evidence-based studies of the use of Foley catheters as gastrostomy tubes [7]. The reasons why Foley catheters are used include the low cost, their small size, which is suitable for infants, their ease of application and clinicians’ familiarity with Foley catheters. In emergency situations, as with our patient, a Foley catheter can be used to ensure that the stoma is patent and that patients have access to fluids, nutrition and medication [4]. However, to eliminate the chances of tube migration, an external fixation device should be applied over the Foley catheter. Securing these tubes properly to the abdominal wall is necessary not only to prevent dislodgment but also to prevent internal migration. The external length should be recorded during tube placement to help identify instances of displacement [11, 17, 22]. Other preventative options include the gastrostomy being placed away from the pylorus [14]. If the catheter is no being longer used, it should be removed to prevent migration [16]. Caregivers should regularly inspect the gastrostomy site and the external length of the tube [11].

In conclusion, patients with ineffective feeding catheters, respiratory tract infections and complaints of vomiting should be evaluated for intestinal obstruction. Awareness of feeding catheter migration in the intestine and its potential to cause intussusception facilitates early diagnosis and treatment. The use of Foley catheters is a temporary measure to avoid closure of the gastrostomy while waiting for proper gastrostomy tube replacements, and they are not recommended as a long-term replacement feeding tube. Therefore, it should be replaced as soon as a gastrostomy tube is available.

## Data Availability

Data and materials will be available upon request.

## Abbreviations

PEG: Percutaneous endoscopic gastrostomy
UAXR: Upright abdominal X-ray
US: Ultrasonography

## References

[1] Chapman A, George K, Selassie A, Lesher AP, Ryan RM (2021). NICU infants who require a feeding gastrostomy for discharge. J Pediatr Surg.

[2] Kumbhar SS, Plunk MR, Nikam R, Boyd KP, Thakrar PD (2020). Complications of percutaneous gastrostomy and gastrojejunostomy tubes in children. Pediatr Radiol.

[3] Hussain M, Thambidorai CR (2000). Intussusception as a complication of gastrostomy tube: a case report. Med J Malays.

[4] Ojo O (2014). Problems with use of a Foley catheter in enteral tube feeding. Br J Nurs.

[5] Bagrodia N, Chong HS, Hoballah J, Scott-Conner C, Chong H (2017). Open (Stamm) gastrostomy. Operative dictations in general and vascular surgery.

[6] Anselmo CB, Tercioti Junior V, Lopes LR, Coelho Neto JdeS, Andreollo NA. Surgical gastrostomy: current indications and complications in a university hospital. Rev Col Bras Cir. 2013;40(6):458–462. 10.1590/s0100-69912013000600007. 10.1590/s0100-69912013000600007 24573623

[7] Gray C, Grobelna A (2019). Urinary catheters as replacement feeding tubes: a review of clinical effectiveness, cost-effectiveness, and guidelines.

[8] Kiatipunsodsai S (2015). Gastrostomy tube replacement using Foley's catheters in children. J Med Assoc Thai.

[9] Gowen GF (1988). The management of complications of Foley feeding gastrostomies. Am Surg.

[10] Bankhead R, Boullata J, Brantley S (2009). Enteral nutrition practice recommendations. JPEN J Parenter Enteral Nutr.

[11] Boullata JI, Carrera AL, Harvey L (2017). ASPEN safe practices for enteral nutrition therapy [Formula: see text]. JPEN J Parenter Enter Nutr.

[12] Khan YA, Yadav SK, Elkholy A (2017). Waugh's syndrome: report of two children with intussusception. Eur J Pediatr Surg Rep.

[13] Kakiuchi T, Nakayama A, Nojiri J, Yamanouchi T, Matsuo M (2020). Jejuno-jejunal intussusception caused by a percutaneous endoscopic gastrojejunostomy tube in a pediatric patient: a case report. Medicine.

[14] Haws EB, Sieber WK, Kiesewetter WB (1966). Complications of tube gastrostomy in infants and children. 15-year review of 240 cases. Ann Surg.

[15] Hughes UM, Connolly BL, Chait PG, Muraca S (2000). Further report of small-bowel intussusceptions related to gastrojejunostomy tubes. Pediatr Radiol.

[16] Ragunath K, Roberts A, Senapati S, Clark G (2004). Retrograde Jejunoduodenal intussusception caused by a migrated percutaneous endoscopic gastrostomy tube. Dig Dis Sci.

[17] Ibegbu E, Relan M, Vega KJ (2007). Retrograde jejunoduodenogastric intussusception due to a replacement percutaneous gastrostomy tube presenting as upper gastrointestinal bleeding. World J Gastroenterol.

[18] Pelosof L, Ringold DA, Kuo E, Bhalla S, Whinney R, Zuckerman GR (2007). Retrograde jejunogastric intussusception caused by a migrated gastrostomy tube. Endoscopy.

[19] Hui GC, Gerstle JT, Weinstein M, Connolly B (2004). Small-bowel intussusception around a gastrojejunostomy tube resulting in ischemic necrosis of the intestine. Pediatr Radiol.

[20] Galea MH, Mayell MJ (1988). Gastroduodenal mucosal intussusception causing gastric outlet obstruction: a complication of gastrostomy tubes. J Pediatr Surg.

[21] Funaki B (2010). Enteroenteric intussusception due to balloon-retained gastrostomy catheter. Semin Intervent Radiol.

[22] Friedman JN (2004). Enterostomy tube feeding: The ins and outs. Paediatr Child Health.

